# Host–microbiome interactions in leukemia: mechanisms, treatment response, and clinical implications

**DOI:** 10.3389/fcimb.2026.1842279

**Published:** 2026-07-08

**Authors:** Jhommara Bautista, Rudy Carrión-Ruiz, Arianna Bourne-Cabezas, Luis Ayala Robles, Ana Maria Velez-Navarrete, Andrés López-Cortés

**Affiliations:** 1 Cancer Research Group (CRG), Faculty of Medicine, Universidad de Las Américas, Quito, Ecuador; 2 Hospital de Especialidades Carlos Andrade Marín, Quito, Ecuador; 3 Sociedad Ecuatoriana de Hematología, Quito, Ecuador

**Keywords:** epithelial barrier integrity, hematopoietic homeostasis, immune function, leukemia, microbiome

## Abstract

Host-microbiome interactions regulate immune function, epithelial barrier integrity, and hematopoietic homeostasis. Intestinal microbial communities show consistent disruption in leukemia, particularly during intensive chemotherapy and hematopoietic stem cell transplantation. Reduced microbial diversity, depletion of short-chain fatty acid (SCFA)–producing commensals, and expansion of opportunistic taxa are recurrent findings across cohorts. Such patterns correlate with inflammatory signaling, impaired barrier function, and shifts in immune responses affecting treatment tolerance and hematopoietic recovery. Clinical associations show greater consistency for treatment-related outcomes, including infection risk, mucosal injury, and delayed immune reconstitution, than for leukemogenesis. Evidence supporting a direct causal role of specific microbial taxa in disease initiation remains limited. This review examines microbiome composition, microbial taxa, and mechanistic pathways in leukemia, with emphasis on how microbiome alterations may influence leukemia biology, disease progression, treatment response, and clinical outcomes, while acknowledging that most human evidence remains associative.

## Introduction

Leukemia comprises malignant disorders arising from hematopoietic precursor cells, characterized by uncontrolled proliferation, impaired differentiation, and disruption of normal blood formation. Leukemia includes biologically distinct entities classified according to disease tempo and hematopoietic lineage, including acute myeloid leukemia, acute lymphoblastic leukemia, chronic lymphocytic leukemia, and chronic myeloid leukemia. Differences in cellular origin, molecular alterations, immune context, treatment exposure, and clinical behavior are relevant for microbiome research, because the biological meaning of microbial alterations may differ across leukemia subtypes and therapeutic settings (Estcourt and Bain, 2013; Achir et al., 2024).

Although leukemogenesis has traditionally been examined through genetic, epigenetic, and microenvironmental mechanisms, increasing attention has turned to systemic regulators of hematopoiesis, including signals derived from the intestinal microbiome. Experimental studies indicate that microbial communities can influence hematopoietic stem and progenitor cell activity through interferon signaling, pattern-recognition receptor pathways, and microbiota-derived metabolites (Bautista et al., 2025b). Short-chain fatty acids (SCFAs), particularly butyrate, participate in epigenetic regulation and cytokine signaling, while microbial activation of interferon pathways has been linked to hematopoietic stem cell quiescence and activation states (Yan et al., 2022).

Microbial effects relevant to leukemia are unlikely to depend only on taxonomic composition. Barrier integrity, microbial product translocation, circulating metabolites, and immune-mediated communication with the bone marrow microenvironment may connect intestinal ecology with hematopoietic regulation. Loss of epithelial barrier function can facilitate systemic exposure to microbial products, amplify inflammatory signaling, and alter hematopoietic output. In parallel, changes in metabolite availability and immune tone suggest that host–microbiome interactions may shape hematopoiesis through integrated inflammatory and metabolic pathways (Zheng et al., 2024; Agarwal et al., 2025; Bautista and López-Cortés, 2026a). This systems-level interpretation is consistent with the concept of host-microbiome decoupling, in which disease-associated dysbiosis reflects impaired functional coordination between microbial ecology and host regulatory capacity rather than taxonomic alteration alone (Bautista and López-Cortés, 2026b).

In hematologic malignancies, microbial dysbiosis has been associated with immune dysregulation, altered inflammatory signaling, epithelial injury, and changes in hematopoietic dynamics. Reduced abundance of SCFA–producing taxa and shifts in microbial metabolic output have been linked to modulation of leukemic cell behavior, impaired epithelial integrity, and sustained inflammatory signaling relevant to disease progression (Wang et al., 2022). Treatment adds a further layer of complexity. Chemotherapy, antibiotics, immunosuppression, and hospitalization can reshape microbial communities, with reported associations with infectious complications, immune recovery, treatment tolerance, and clinical outcomes in leukemia and related hematologic conditions (Jalalifar et al., 2026).

Microbiome alterations in leukemia should be interpreted within the biological and clinical context in which they arise. Dysbiosis may reflect disease-associated immune disruption, treatment-related injury, antibiotic exposure, hospitalization, or broader metabolic stress; however, experimental and clinical findings also suggest that microbial metabolites, epithelial barrier dysfunction, immune signaling, and gut–bone marrow communication may influence hematopoietic regulation, leukemic progression, treatment tolerance, and prognosis. Current evidence remains heterogeneous, and longitudinal studies are still insufficient to define whether microbial changes precede leukemogenesis, accompany disease evolution, or persist after therapy. Therefore, the microbiome is best considered a dynamic component of the leukemia ecosystem rather than an isolated causal factor, with functional relevance depending on disease subtype, treatment phase, host immunity, and methodological context (Johansson et al., 2025; Zhang et al., 2024; Gust et al., 2017).

Longitudinal studies remain limited in leukemia, particularly for microbial changes that precede diagnosis, accompany disease progression, or persist after treatment. Microbiome alterations should therefore be interpreted with caution and not as a defined temporal sequence. This review focuses on evidence exploring how host–microbiome interactions may influence leukemia development, disease progression, treatment response, and therapy-related complications through dysbiosis, immune-metabolic disruption, epithelial barrier dysfunction, and altered gut–bone marrow communication. Given that most clinical studies remain observational, microbiome alterations are discussed as disease-associated features unless supported by experimental or longitudinal evidence suggesting functional involvement.

## Microbiome composition and dysbiosis across leukemia subtypes

In healthy individuals, the intestinal microbiome forms a complex microbial ecosystem involved in metabolic regulation, epithelial barrier maintenance, and immune homeostasis. Dominant bacterial phyla typically include Firmicutes, Bacteroidetes, Actinobacteria, and Proteobacteria, which participate in nutrient metabolism and the production of microbial metabolites such as SCFAs. Metabolites including butyrate, acetate, and propionate influence epithelial integrity, regulatory immune pathways, and inflammatory signaling, thereby contributing to stable host-microbe interactions under physiological conditions (Jalalifar et al., 2026; Goswami and Bose, 2024; Bautista and López-Cortés, 2025).

Microbial metabolic activity has been linked to systemic immune regulation and hematopoietic processes. Microbial metabolites are associated with modulation of immune cell differentiation, cytokine production, and inflammatory balance, and may interact with the bone marrow microenvironment to influence hematopoietic regulation through immune and metabolic pathways (Yang et al., 2024). Within this biological context, intestinal microbial communities constitute an environmental component capable of interacting with hematologic regulation.

Alterations in microbial composition have been reported across different leukemia subtypes, but their biological interpretation depends on disease lineage, disease course, age distribution, treatment exposure, and immune status. Microbiome alterations in leukemia should therefore not be interpreted as a uniform phenomenon. Interpreting the evidence by leukemia subtype helps separate recurrent microbial patterns from changes that are more closely related to specific disease contexts (Pötgens et al., 2023) (**Table 1**).

**Table 1:** Subtype-specific microbiome features and mechanistic patterns in leukemia.

Leukemia subtype	Main microbial pattern	Mechanistic interpretation	Clinical relevance	Current limitation	Key references
AML	Reduced gut microbial diversity, depletion of SCFA-producing anaerobes, altered butyrate profiles, and enrichment of opportunistic taxa in some cohorts.	Barrier dysfunction, endotoxin exposure, inflammatory signaling, and possible gut–bone marrow crosstalk.	Infectious risk during induction chemotherapy, treatment-related complications, and hematologic recovery.	Causality remains unresolved because disease biology, chemotherapy, antibiotics, and hospitalization overlap.	(Galloway-Peña et al., 2020; Wang et al., 2022)
ALL	Chemotherapy-associated dysbiosis, reduced diversity, incomplete microbiome recovery, and enrichment of Proteobacteria, Enterococcaceae, or Streptococcaceae in high-risk contexts.	Mucosal injury, impaired colonization resistance, inflammatory activation, and delayed immune-microbial recovery.	Pediatric chemotherapy toxicity, febrile neutropenia, infection susceptibility, and microbiome restoration after treatment.	Interpretation is strongly affected by age, antibiotics, diet, chemotherapy intensity, and microbiome maturation.	(Hakim et al., 2018)
CLL	Lower microbial diversity and heterogeneous inflammatory microbial configurations.	Possible interaction with chronic immune dysregulation and altered inflammatory tone.	Immune status, infection susceptibility, and clinical heterogeneity.	Small cohorts; no validated CLL-specific microbial signature.	(Faitova et al., 2024)
CML	Limited subtype-specific evidence; microbial variation has mainly been discussed within broader leukemia cohorts.	Possible immune-metabolic modulation, but no direct CML-specific mechanism has been established.	Treatment tolerance, inflammatory status, and long-term host physiology.	Evidence is insufficient; findings should not be extrapolated from acute leukemia.	(Zhou et al., 2022)

### Acute myeloid leukemia

In acute myeloid leukemia (AML), available evidence indicates that gut microbial disruption is not limited to treatment exposure. Metagenomic and metabolomic studies have identified altered microbial composition at diagnosis, including reduced representation of commensal anaerobes and changes in microbial metabolic pathways. These alterations have been associated with systemic manifestations such as anorexia, muscle weakness, and reduced functional status, suggesting that the gut microbiome may reflect disease-related metabolic stress before intensive therapy (An et al., 2025). Mechanistically, AML is the leukemia subtype in which the microbiome–metabolome axis has been most clearly linked to disease biology. Reduced abundance of butyrate-producing taxa, particularly *Faecalibacterium*, has been associated with impaired epithelial integrity, increased endotoxin exposure, inflammatory activation, and changes in leukemic burden or treatment-related recovery (Wang et al., 2022). Higher microbial diversity and enrichment of commensal anaerobes have also been associated with improved hematologic recovery after induction therapy, whereas disrupted community structure aligns with impaired regenerative capacity (Tian et al., 2025). However, current human evidence remains largely associative, and experimental support is stronger for modulation of disease progression, inflammatory signaling, and treatment recovery than for leukemia initiation.

### Acute lymphoblastic leukemia

In acute lymphoblastic leukemia (ALL), most available microbiome evidence comes from pediatric cohorts, where interpretation requires consideration of age-dependent microbiome maturation, chemotherapy exposure, antibiotic use, nutritional changes, and mucosal injury. A recurrent pattern is reduced microbial diversity during therapy, incomplete recovery of commensal populations, and depletion of bacteria involved in butyrate production and epithelial homeostasis. Compared with AML, evidence in ALL is more consistently linked to treatment-related complications than to direct leukemic biology. Microbial imbalance has been associated with oral and intestinal mucositis, infectious susceptibility, inflammatory cytokine profiles, gastrointestinal toxicity, and delayed ecological recovery after chemotherapy. In ALL, the microbiome therefore appears most relevant as a modifier of treatment tolerance, immune recovery, and mucosal complications, rather than as a defined driver of leukemogenesis.

### Chronic lymphocytic leukemia

In chronic lymphocytic leukemia (CLL), microbiome research remains more limited, but available studies indicate reduced gut microbial diversity and enrichment of inflammatory microbial configurations associated with clinical heterogeneity. Unlike acute leukemias, CLL develops within a prolonged state of immune dysregulation, making it difficult to determine whether dysbiosis contributes to disease progression or reflects chronic alterations in host immunity. A cautious interpretation is that gut microbial imbalance may interact with systemic immune dysfunction, inflammation, and host vulnerability. At present, available evidence does not support a reproducible CLL-specific microbial signature, but it suggests that microbial diversity and inflammatory microbial patterns may correlate with disease behavior (Faitová et al., 2022). This point is particularly relevant in chronic leukemia contexts because aging-associated microbiome remodeling, barrier dysfunction, inflammaging, and immunosenescence may modify host-microbiome associations independently of leukemia biology (Bautista and López-Cortés, 2026c).

### Chronic myeloid leukemia

Evidence in chronic myeloid leukemia (CML) remains comparatively scarce. Available analyses suggest that gut microbial variation may differ across leukemia subtypes, including CML, but subtype-specific mechanisms are not yet well established (Guevara-Ramírez et al., 2023). Because CML is primarily driven by a defined oncogenic lesion and is commonly treated with targeted kinase inhibition, microbiome-related effects may be more relevant to immune modulation, treatment tolerance, metabolic status, or long-term host physiology than to disease initiation (Caprino et al., 2026). Future CML-specific studies should evaluate whether microbial profiles influence inflammatory state, therapeutic tolerance, adverse events, or depth of molecular response, rather than extrapolating mechanistic conclusions from AML or ALL.

Across leukemia subtypes, the most reproducible finding is ecological disruption rather than a single leukemia-specific microbial signature. Reduced diversity, depletion of obligate anaerobic commensals, loss of SCFA-producing taxa, altered microbial metabolic output, and expansion of opportunistic organisms recur across several clinical contexts. However, their interpretation differs by subtype. In AML, microbial disruption is most closely linked to metabolic dysfunction, epithelial barrier impairment, endotoxin exposure, inflammatory signaling, and hematologic recovery. In pediatric ALL, dysbiosis is more strongly associated with chemotherapy-related mucosal injury, infection susceptibility, and delayed microbiome recovery. In CLL, microbial alterations appear to intersect with chronic immune dysregulation and clinical heterogeneity. In CML, current evidence remains insufficient to define a distinct microbiome-associated mechanism. This subtype-based synthesis supports a cautious interpretation grounded in subtype-specific biology and avoids overgeneralization across clinically and molecularly distinct diseases.

Treatment exposure adds an additional layer of interpretation to subtype-specific microbiome findings. Intensive chemotherapy, broad-spectrum antibiotics, mucosal injury, hospitalization, nutritional changes, and hematopoietic cell transplantation can reshape microbial ecology independently of leukemia biology. These factors may reduce microbial diversity, deplete obligate anaerobic commensals, favor expansion of opportunistic organisms, and alter microbial metabolic output. Therefore, microbiome alterations observed during leukemia care should be interpreted according to disease subtype, treatment phase, antimicrobial exposure, and host immune status. Longitudinal microbiome profiling integrated with metabolomic, immune, and clinical data will be required to clarify whether microbial ecosystems contribute to leukemia progression and therapeutic response, reflect disease- and treatment-related disruption, or act through both pathways (Yang et al., 2024; Ji et al., 2025).

## Microbial taxa associated with leukemia

### Gut microbiome and leukemia

The intestinal microbiome constitutes a complex microbial ecosystem involved in immune regulation, epithelial barrier integrity, and metabolic signaling. In healthy individuals, intestinal microbial communities are typically dominated by members of the phyla Firmicutes and Bacteroidetes, accompanied by smaller proportions of Actinobacteria and Proteobacteria. Microbial populations differ across leukemia cohorts, with depletion of commensal anaerobic genera and expansion of facultative bacteria associated with inflammatory intestinal environments. Through multiple signaling pathways, intestinal microorganisms may influence systemic immune balance and participate in processes related to hematopoietic regulation, although direct causal effects in leukemia patients remain incompletely established (D’Angelo et al., 2021; Vázquez et al., 2024).

Across leukemia studies, the most reproducible finding is not a single disease-defining microbial signature, but a pattern of ecological instability influenced by leukemia subtype, treatment phase, antibiotic exposure, and host immune status. Several cohorts report reduced microbial diversity and depletion of commensal anaerobes, including SCFA-producing taxa such as *Faecalibacterium*, *Blautia*, and *Ruminococcus*. In treatment-intensive settings, particularly induction chemotherapy and hematopoietic cell transplantation, expansion of facultative or opportunistic taxa such as *Enterococcus*, *Streptococcus*, *Escherichia*, and other Proteobacteria is frequently described. Overall, functional disruption appears more consistent than taxonomic reproducibility, with effects involving colonization resistance, epithelial integrity, microbial metabolite availability, and inflammatory signaling (Alvaro et al., 2026; Hakim et al., 2018; Jalalifar et al., 2026).

Subtype-specific interpretation is therefore required. In AML, microbial changes at diagnosis and during induction therapy are most closely linked to metabolic disruption, SCFA depletion, endotoxin exposure, and hematologic recovery (Tian et al., 2025). In pediatric ALL, dysbiosis is more consistently associated with chemotherapy-related mucosal injury, oral and intestinal inflammation, infection risk, and delayed microbiome recovery (Oldenburg et al., 2021). In CLL, available evidence suggests reduced diversity and inflammatory microbial configurations in relation to clinical heterogeneity, although causality remains unclear (Faitová et al., 2022). In CML, subtype-specific evidence remains limited, and microbiome findings should be interpreted cautiously until dedicated cohorts are available (Ye et al., 2025). This interpretation avoids overstatement of taxonomic associations and better reflects the current state of the field.

### Oral microbiome and hematologic malignancies

Microbial communities of the oral cavity represent another ecological niche increasingly examined in hematologic malignancies. Oral bacterial populations form structured biofilms on mucosal surfaces and dental structures. Dominant genera commonly include Streptococcus, with additional representation of Prevotella, Veillonella, Neisseria, and Haemophilus, which together define the core composition of the healthy oral microbiome (Wang et al., 2014). Disruption of this equilibrium becomes evident in hematologic settings characterized by intensive treatment exposure.

Hematopoietic stem-cell transplantation and chemotherapy induce marked alterations in oral microbial ecology, characterized by reduced diversity and shifts in taxonomic composition. Increased abundance of taxa such as Enterococcus and Streptococcus has been reported during periods of mucosal injury, antimicrobial exposure, and immunosuppression, conditions that favor the expansion of microorganisms adapted to inflammatory environments (Sánchez-Becerra et al., 2026; Pereira et al., 2025).

Variation in oral microbial composition is not restricted to treatment phases but has also been linked to clinical outcomes. Differences in the relative abundance of genera including *Streptococcus* and *Veillonella* before transplantation have been associated with subsequent leukemia relapse following allogeneic transplantation, suggesting that baseline microbial states may reflect host vulnerability, contribute to immune conditions permissive for recurrence, or both (Faraci et al., 2024). In parallel, analyses across distinct oral niches such as saliva, tongue, and dental plaque indicate that microbial alterations are spatially heterogeneous, with shifts involving *Enterococcus*, *Streptococcus*, and *Lactobacillus* observed during transplantation and recovery, often in association with mucosal inflammation and infectious complications (Xu et al., 2015; Tamahara et al., 2025).

In ALL, chemotherapy further modifies oral microbial composition, with reduced abundance of commensal taxa and expansion of microorganisms adapted to inflammatory conditions. Such alterations correlate with the severity of oral mucositis and increased concentrations of pro-inflammatory cytokines in saliva, linking microbial imbalance with clinically relevant complications (Wang et al., 2021; Hong et al., 2019). Integration of oral and intestinal microbiome datasets has extended these observations beyond single-site analyses, allowing the identification of multi-site microbial patterns associated with transplantation outcomes. Combined profiling approaches have been incorporated into predictive models evaluating complications such as graft-versus-host disease and treatment-related mortality, although the extent to which these associations reflect causal mechanisms remains to be clarified (Tian et al., 2025).

### Bacterial translocation and systemic inflammation

Microbial alterations associated with leukemia frequently occur alongside epithelial barrier disruption and microbial translocation. Intensive chemotherapy, antimicrobial exposure, and immune dysfunction associated with hematologic malignancies may compromise intestinal epithelial integrity. Under such conditions, microbial components or viable bacteria can cross mucosal barriers and enter systemic compartments (Jalalifar et al., 2026; Song et al., 2020).

Bloodstream infections in patients with hematologic malignancies occur in the context of mucosal barrier injury and immunosuppression. Disruption of epithelial integrity permits translocation of microorganisms, including enteric taxa such as Enterococcus and Enterobacteriaceae, into systemic circulation, where they contribute to bacteremia (Choi et al., 2022).

Microbial molecules released during translocation has been linked to systemic immune signaling. Bacterial components including lipopolysaccharides, peptidoglycans, and other pathogen-associated molecular patterns interact with host pattern-recognition receptors and activate innate immune pathways. Activation of inflammatory signaling cascades may influence cytokine production and contribute to alterations in the hematopoietic microenvironment (Hajishengallis and Chavakis, 2026; Oh et al., 2025).

Chemotherapy and hematopoietic stem-cell transplantation define clinical contexts marked by concurrent epithelial injury and immune suppression. Under these conditions, microbial alterations extend beyond a single anatomical site, involving both intestinal and oral ecosystems, with compositional shifts reported across disease stages and treatment exposure. Impairment of epithelial integrity facilitates microbial translocation, which has been associated with infectious complications and systemic inflammatory responses influencing the hematopoietic environment (Zhang et al., 2025; Zhou et al., 2022).

## Molecular mechanisms linking microbiome and leukemia

Mechanistic interpretation requires separating pathways observed across leukemia contexts from those supported mainly in specific subtypes. The available evidence points to four recurring axes: depletion of SCFA-producing commensals, epithelial barrier disruption, translocation of microbial products, and immune-metabolic signaling affecting the hematopoietic niche. However, support for each mechanism is uneven across leukemia subtypes. In AML, the strongest evidence relates to microbiome–metabolome disruption, including butyrate depletion, endotoxin exposure, inflammatory signaling, and impaired hematopoietic recovery (Alvaro et al., 2026). In ALL, dysbiosis is more consistently linked to chemotherapy-associated mucosal injury, infection susceptibility, oral inflammation, and delayed immune reconstitution (Chen et al., 2023). In CLL, current findings suggest a relationship between microbial diversity and chronic immune dysregulation, whereas CML-specific mechanistic evidence remains insufficient (Yang et al., 2024).

Intestinal microbial ecosystems interact with hematopoietic regulation through epithelial integrity, immune signaling, and systemic inflammatory pathways. Disruption of microbial composition is associated with increased intestinal permeability and sustained exposure to microbial-derived molecules that reach systemic circulation and influence bone marrow function (Liu et al., 2024; Bautista et al., 2025a).

Chronic inflammation and the hematopoietic microenvironment. Leukemia-associated dysbiosis frequently involves depletion of obligate anaerobic microorganisms linked to epithelial maintenance, including taxa responsible for preserving mucosal integrity and metabolic stability. Loss of these microbial functions is associated with barrier impairment and increased permeability, which facilitates translocation of microbial components such as lipopolysaccharide into systemic circulation. Persistent exposure to microbial products has been associated with increased cytokine signaling and changes in the hematopoietic microenvironment, particularly through pathways involved in inflammatory regulation and progenitor cell behavior (Khalil and Maher, 2024).

In AML, reduced abundance of *Faecalibacterium* coincides with decreased butyrate levels, impaired epithelial integrity, and elevated circulating endotoxin. Endotoxin exposure promotes leukemic progression, whereas restoration of *Faecalibacterium* or supplementation with butyrate is associated with delayed disease progression and improved epithelial function. Observations from experimental and clinical settings link intestinal disruption with sustained inflammatory signaling capable of influencing leukemia biology beyond local gastrointestinal effects (Tong and Lou, 2025; Wang et al., 2022). Intestinal dysfunction therefore contributes to a pro-inflammatory systemic environment with measurable effects on leukemic dynamics.

Comparable patterns are reported in CLL and pediatric leukemia. Reduced microbial diversity correlates with less favorable disease behavior in CLL, while pediatric cohorts exhibit increased infection susceptibility, gastrointestinal toxicity, and delayed microbiome recovery following treatment. Repeated epithelial injury, antimicrobial exposure, and persistent inflammatory activation progressively modify marrow-directed signaling and reduce stability within the hematopoietic niche (Faitova et al., 2024; Roganovic et al., 2025).

### Immune modulation by the microbiome

Alterations in microbial composition may influence immune regulation across innate and adaptive compartments, particularly through changes in antigen exposure, metabolite availability, epithelial barrier integrity, and inflammatory signaling. Changes in antigen exposure, cytokine gradients, and immune cell differentiation contribute to a dysregulated immune environment that permits leukemic persistence. Comparable cancer models indicate that microbiome-associated immune dysregulation can converge with suppressive microenvironmental programs that limit effective antitumor immunity. In acute leukemia, depletion of anti-inflammatory commensals combined with enrichment of proinflammatory microbial signatures correlates with sustained immune activation without effective tumor control (Li et al., 2024; Bautista et al., 2026d).

In CLL, reduced microbial diversity and enrichment of inflammatory bacterial patterns have been associated with clinical heterogeneity, suggesting that intestinal microbiota–linked immune perturbations may contribute to, accompany, or reflect differences in disease progression across patients. Pediatric leukemia adds another layer to the same framework, as microbial imbalance has been linked to impaired mucosal homeostasis, greater infectious burden, and altered immune recovery during therapy. Such observations support the view that gut-derived immune perturbations are not restricted to intestinal complications and may shape host-tumor interactions more broadly (Faitova et al., 2024).

Interventional data further support a functional role of the microbiome in immune recovery. In AML, autologous fecal microbiota transfer following intensive chemotherapy restores microbial diversity and reduces the abundance of antibiotic resistance genes, indicating partial reconstruction of the intestinal ecosystem after treatment-induced disruption (Malard et al., 2021). Independent observations in hematologic malignancies show that gut microbial composition correlates with treatment efficacy and toxicity, indicating that microbial ecological configuration modulates clinical tolerance and therapeutic response (Uribe-Herranz et al., 2021).

### Gut-bone marrow axis in leukemogenesis

The gut-bone marrow axis integrates inflammatory, metabolic, and immune pathways linking intestinal ecology with hematopoietic regulation. Under physiological conditions, microbial activity contributes to epithelial stability, immune balance, and controlled hematopoietic output. Disruption of microbial equilibrium leads to altered metabolite availability, increased permeability, and sustained inflammatory signaling, modifying molecular cues reaching marrow progenitors and stromal compartments. Leukemia-associated dysbiosis may represent both a consequence of disease- and treatment-related disruption and a source of systemic regulatory disturbance with potential functional consequences for marrow biology (Liu et al., 2024; Wells et al., 2024). Differential associations between specific gut microbial taxa and leukemia risk were observed across ALL, AML, CML, and CLL, indicating that variation in intestinal microbial composition is systematically related to susceptibility to distinct hematologic malignancies (Chen et al., 2023).

In AML, enrichment of commensal anaerobes and higher microbial diversity correlates with improved hematologic recovery following induction therapy, whereas disrupted community structure aligns with impaired regenerative dynamics (Salvestrini et al., 2025). Microbial imbalance, altered metabolite production, epithelial barrier disruption, and immune dysregulation intersect with hematopoietic control and influence both leukemia progression and treatment response (Hajjar et al., 2026).

### Common and subtype-specific mechanistic patterns

Evidence from leukemia cohorts favors a pattern-based interpretation of the microbiome rather than a taxon-centered one. Across AML, ALL, CLL, and transplant-related settings, the recurrent finding is ecological disruption, reflected by reduced diversity, loss of anaerobic commensals, impaired microbial metabolic output, and expansion of organisms favored by inflammation, antibiotic exposure, or mucosal injury (Yang et al., 2024). The subtype-specific signal varies by clinical context. AML shows the clearest relationship with metabolic and inflammatory mechanisms, including SCFA depletion, barrier dysfunction, endotoxin exposure, and altered hematopoietic recovery. ALL, particularly in pediatric cohorts, is more closely associated with treatment-induced dysbiosis, mucosal injury, oral inflammation, infectious complications, and delayed immune recovery. CLL appears to intersect with chronic immune dysregulation and reduced microbial diversity, although the evidence remains limited. For CML, current data are insufficient to define a distinct microbiome-associated mechanism (Faitova et al., 2024; Jalalifar et al., 2026; Alvaro et al., 2026).

At the mechanistic level, these observations point to a limited group of recurrent pathways. Depletion of SCFA-producing bacteria may compromise epithelial integrity and reduce anti-inflammatory signaling. Barrier damage may facilitate microbial product translocation and activation of TLR- and NOD-dependent pathways. Systemic exposure to microbial products may amplify IL-6, TNF, interferon-related signaling, and inflammatory cues affecting bone marrow function. Altered microbial metabolism may also influence hematopoietic recovery and treatment tolerance. Overall, microbiome alterations in leukemia are better interpreted as functional ecological states shaped by disease subtype, treatment phase, and host immune status, rather than as isolated bacterial signatures (Gawronski et al., 2026; Neoman et al., 2025; Wang et al., 2022).

## Microbiome-metabolome interactions in leukemia

### Microbial metabolites in hematopoietic regulation and leukemia progression

Microbiome-derived metabolites regulate hematopoiesis through coordinated effects on immune signaling, metabolic homeostasis, and bone marrow niche function. Altered metabolite profiles described in leukemia include reduced availability of compounds involved in immunomodulation and epithelial support, accompanied by reconfiguration of cytokine networks regulating hematopoietic stem and progenitor cell (HSPC) quiescence, self-renewal, and lineage commitment. Perturbation of signaling axes involving interleukin-6, tumor necrosis factor, and interferon pathways has been linked to skewed myelopoiesis and sustained inflammatory tone, reflecting metabolic contributions to altered hematopoietic output. These patterns support a gut-bone marrow axis in which ecological imbalance corresponds to functional changes in hematopoiesis rather than isolated taxonomic variation (Zhang et al., 2022; Baumann and Ellegast, 2025; Maeda et al., 2009).

SCFA, such as butyrate, constitute a central mechanistic component. Reduced abundance of butyrate-producing taxa parallels depletion of metabolic functions associated with immune regulation and transcriptional control. In AML, lower representation of butyrate-associated genera correlates with increased leukocyte counts, higher blast burden, and less favorable-risk profiles, linking metabolite depletion with more aggressive disease states (Wang et al., 2022; Perl et al., 2025). At the molecular level, butyrate regulates histone acetylation through inhibition of histone deacetylases, modulates NF-κB–dependent inflammatory signaling, and influences transcriptional programs controlling proliferation, apoptosis, and differentiation. Reduced availability alters HSPC output and leukemic cell behavior through combined epigenetic and inflammatory mechanisms (Zhang et al., 2026; Hinterbrandner et al., 2024).

Disruption of microbial metabolic output also affects therapeutic response. Dysbiotic configurations associated with chemotherapy resistance in AML have been linked to altered metabolite availability, indicating that metabolic context contributes to leukemic persistence under treatment pressure. Longitudinal microbiome-metabolome analyses show that ecological instability during therapy coincides with dynamic shifts in metabolite composition, supporting integration of taxonomic and functional readouts for clinical interpretation. Microbial metabolites therefore represent functional parameters for patient stratification and potential targets for metabolic modulation strategies aimed at improving treatment response in leukemia (Tian et al., 2025; Cai et al., 2025).

Metabolomic alterations in leukemia patients. Leukemia patients exhibit metabolic profiles consistent with loss of beneficial microbial functions. In newly diagnosed AML, gut dysbiosis coincides with reduced fecal SCFA concentrations, indicating impaired microbial metabolic output beyond taxonomic variation (An et al., 2025). Alterations are detectable at diagnosis prior to intensive treatment, indicating that intestinal dysfunction emerges early in disease development.

Microbiome configurations in AML differ from patterns observed in healthy individuals and show correlations with clinical features such as muscle weakness and anorexia, supporting a link between intestinal dysfunction and systemic manifestations of disease (Pötgens et al., 2024). Integrative analyses indicate that metabolomic profiling provides a more direct representation of microbiome-associated biological activity than taxonomic composition alone, particularly when distinct microbial communities produce overlapping metabolic outputs (Dumitru et al., 2025).

Treatment-related factors further intensify metabolic disruption. Chemotherapy, broad-spectrum antibiotics, nutritional limitation, and hospitalization reduce microbial diversity and destabilize intestinal ecology, affecting production of metabolites involved in mucosal repair and immune regulation (Vázquez et al., 2024). Associations between gut microbiome features and hematologic recovery after induction therapy indicate that restoration of microbial metabolic function parallels marrow regeneration in a subset of patients (Xu et al., 2023).

Metabolite-mediated molecular pathways. Microbial metabolites influence leukemia biology through a restricted set of mechanisms supported by experimental and clinical evidence. In AML, reduced abundance of SCFAs, particularly butyrate, has been associated with impaired intestinal barrier integrity and increased systemic exposure to microbial products. Barrier disruption permits translocation of lipopolysaccharide, leading to activation of TLR4–MyD88–NF-κB signaling and induction of pro-inflammatory cytokines, including interleukin-6 and tumor necrosis factor. Sustained activation of this pathway has been linked to leukemic progression and impaired hematopoietic recovery (Mao et al., 2025; Goswami and Bose, 2024).

Beyond barrier-associated mechanisms, microbiota-derived signals regulate hematopoiesis through immune and stromal interactions within the bone marrow microenvironment. Microbial ligands and metabolites influence cytokine production by mesenchymal stromal cells and other niche components, modulating hematopoietic stem and progenitor cell function. Evidence supports involvement of pattern recognition receptor pathways, including NOD1-dependent signaling, in the regulation of hematopoietic activity and systemic immune tone (Liu et al., 2024; Cheng et al., 2023).

Epigenetic regulation represents an additional mechanism. SCFAs act as histone deacetylase (HDAC) inhibitors, altering chromatin accessibility and transcriptional programs associated with proliferation, apoptosis, and immune differentiation. Effects extend to hematopoietic progenitor cell differentiation, linking microbial metabolism with gene regulation in hematopoietic compartments (Farhadipour et al., 2023).

Microbial metabolites influence antileukemic immunity in clinical settings. In hematopoietic stem cell transplantation, metabolite availability is associated with modulation of immune reconstitution and graft-versus-leukemia activity through effects on T-cell function and inflammatory balance. Dysbiosis-associated metabolic states correlate with treatment response and toxicity, indicating that microbial metabolism contributes to therapeutic variability (Burk and Apostolova, 2024; Bautista et al., 2026a).

Therapeutic potential of microbiome-derived metabolites. Microbial metabolites are being evaluated as biomarkers and therapeutic targets in leukemia. SCFAs represent the most consistent candidates due to their association with epithelial repair, immune regulation, and hematopoietic signaling. Reduced SCFA levels in AML support the concept that restoration of microbial metabolic function constitutes a relevant therapeutic objective, either through preservation of beneficial microbial communities or recovery of key biochemical pathways affected by dysbiosis (Galloway-Peña et al., 2020; An et al., 2025).

Additional interest involves bile acid metabolism, tryptophan-derived metabolites, and other microbial products capable of modulating host pathways linked to disease progression and treatment tolerance (Perl et al., 2025). Functional readouts derived from microbial metabolism contribute to patient stratification, monitoring of ecological recovery, and optimization of supportive care during chemotherapy, transplantation, or cellular therapy. Associations between microbiome features and hematologic recovery further support the clinical relevance of microbial metabolic outputs beyond descriptive taxonomic profiling (Jalalifar et al., 2026).

## Microbiome-targeted therapeutic strategies in leukemia

Microbiome disruption and treatment outcomes during chemotherapy. Chemotherapy for leukemia disrupts the intestinal microbiota; however, the clinical relevance of these changes depends on how the resulting microbial state may affect treatment tolerance, infectious risk, epithelial injury, and hematopoietic recovery. Recurrent findings across adult and pediatric settings include reduced microbial diversity, depletion of obligate anaerobes that sustain colonization resistance, and expansion of taxa favored by inflammation, hospitalization, and antibiotic exposure. In adults with AML receiving induction therapy, pretreatment and early-treatment microbial configurations have been associated with subsequent infectious complications, suggesting that the microbiome may help identify patients at greater vulnerability during neutropenia. In children with ALL, sequential sampling has shown persistent ecological instability and incomplete recovery of commensal populations during chemotherapy, indicating that microbial injury may extend beyond the immediate treatment phase and affect post-treatment recovery (Liu et al., 2020; Chua et al., 2020).

Clinical relevance is not limited to compositional change. Mucosal injury, prolonged neutropenia, nutritional restriction, and repeated administration of broad-spectrum antibiotics coincide with depletion of barrier-supporting bacteria and enrichment of organisms adapted to inflammatory conditions. Treatment-associated alterations may compromise epithelial integrity, reduce colonization resistance, and facilitate microbial translocation. The baseline or early-treatment microbiome may also shape the magnitude of these complications, contributing to interpatient variability in toxicity and recovery (Masetti et al., 2021; Guevara-Ramírez et al., 2024).

**Figure 1:**
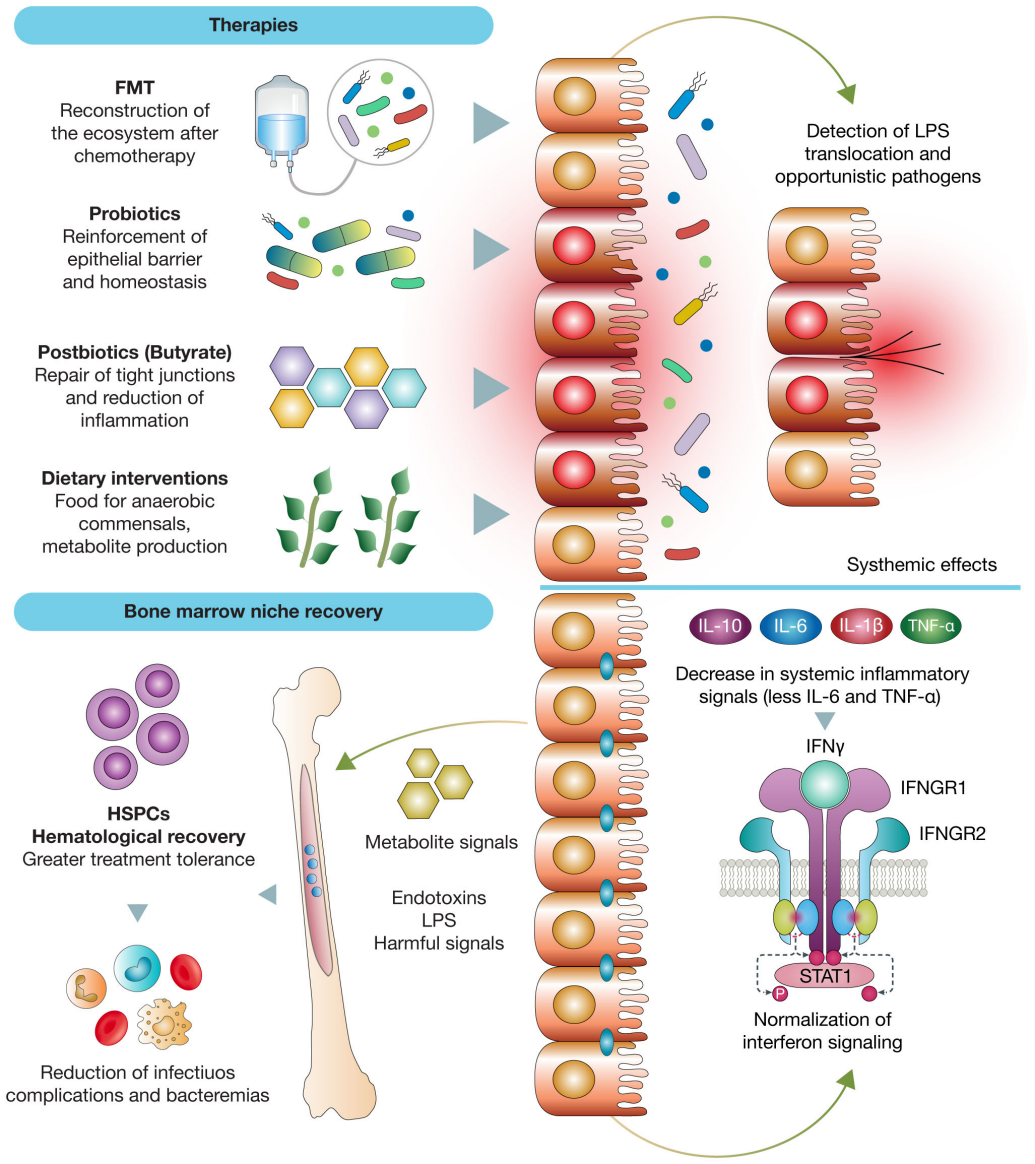
Microbiome-targeted therapeutic strategies in leukemia. Chemotherapy- and antibiotic-associated dysbiosis in leukemia disrupts intestinal microbial ecology, impairs epithelial barrier integrity, promotes translocation of LPS and opportunistic pathogens, and amplifies systemic inflammatory signaling. Microbiome-directed interventions, including FMT, probiotics, postbiotics such as butyrate, and dietary interventions, may help reconstruct microbial communities, reinforce epithelial homeostasis, restore tight-junction integrity, and reduce inflammation. These effects are associated with lower levels of systemic inflammatory mediators, including IL-6 and TNF-α, and with normalization of interferon signaling through the IFNγ–STAT1 axis. In parallel, restoration of gut microbial function may improve gut–bone marrow communication by reducing endotoxin exposure and harmful inflammatory cues while re-establishing beneficial metabolite signaling to the hematopoietic niche. At the bone marrow level, these changes may support HSPC recovery, improve treatment tolerance, and reduce infectious complications and bacteremia. The figure summarizes a conceptual model in which microbiome restoration contributes to both intestinal and systemic recovery in leukemia care. FMT, fecal microbiota transplantation; LPS, lipopolysaccharide; HSPCs, hematopoietic stem and progenitor cells; IFNγ, interferon gamma; IFNGR1, interferon gamma receptor 1; IFNGR2, interferon gamma receptor 2; STAT1, signal transducer and activator of transcription 1; IL-10, interleukin-10; IL-6, interleukin-6; IL-1β, interleukin-1 beta; TNF-α, tumor necrosis factor alpha.

Probiotics and microbiome restoration. Probiotics have been considered as a strategy to limit chemotherapy-associated dysbiosis and support microbiome recovery. Proposed benefits include reinforcement of epithelial barrier function, partial restoration of colonization resistance, attenuation of inflammation-associated microbial shifts, and recovery of metabolites relevant to mucosal and immune homeostasis. In leukemia-oriented literature, strains within *Lactobacillus* and *Bifidobacterium* are frequently discussed because treatment exposure often reduces bacterial groups associated with intestinal stability and immune regulation (Jalalifar et al., 2026; Zhang et al., 2024) (**Figure 1**).

Routine probiotic administration is not universally supported in patients with leukemia. Extensive mucosal injury and sustained neutropenia permit loss of epithelial containment, increasing the likelihood that administered microorganisms translocate beyond the intestinal lumen. This concern is particularly relevant in pediatric leukemia and transplant-adjacent settings, where epithelial vulnerability and immunosuppression are often pronounced. Therapeutic value therefore depends on careful selection of strain, timing of administration, antimicrobial co-exposure, and host condition rather than on supplementation alone (Martyniak et al., 2023; Šimiaková and Bielik, 2024).

An unresolved issue concerns the distinction between taxonomic and functional recovery. Detection of administered probiotic organisms does not ensure reestablishment of microbial activities relevant to leukemia care, including SCFA production, preservation of epithelial integrity, and regulation of pathobiont expansion. A functional perspective is therefore more informative than a purely compositional one, especially when the therapeutic objective is reduction of toxicity, preservation of barrier function, and improvement of treatment tolerance (Guevara-Ramírez et al., 2024; Nowicka et al., 2025).

Fecal microbiota transplantation. Fecal microbiota transplantation (FMT) provides a broader restorative option when chemotherapy and antibiotic-associated dysbiosis exceeds what single-strain supplementation is likely to correct. Its main value lies in reintroducing a complex donor-derived microbial community capable of restoring diversity, metabolic redundancy, and colonization resistance. In hematologic settings, FMT has been explored in recurrent *Clostridioides difficile* infection, severe post-antibiotic dysbiosis, colonization by multidrug-resistant organisms, and complications associated with hematopoietic cell transplantation (Bou Zerdan et al., 2022; van Lier et al., 2023).

Evidence from interventional work indicates that FMT can restore microbial diversity and increase donor-associated commensal taxa after major ecological collapse. In allogeneic hematopoietic cell transplantation and AML, oral third-party FMT improved intestinal dysbiosis, increased beneficial commensals, and reduced pathobiont abundance. Infection rates, however, were not significantly reduced across all measured endpoints. The observed dissociation indicates that compositional recovery and clinical benefit do not necessarily proceed in parallel, and that ecological restoration alone may be insufficient to influence outcomes shaped by multiple treatment-related factors (Rashidi et al., 2023; Habibi and Rashidi, 2023).

Use of FMT in leukemia therefore appears more justifiable in selected scenarios than as a routine adjunct. Interpretation depends on rigorous donor screening, route of administration, antibiotic timing, depth of immunosuppression, and the possibility of transferring undesirable organisms or microbial functions. Current evidence supports clinical interest where dysbiosis is severe and persistent, but broader incorporation into leukemia care will require stronger standardization and clearer identification of patients most likely to benefit (Malard et al., 2022; Wardill et al., 2019).

Microbiome engineering and bacterial therapeutics. More recent approaches aim to move beyond broad ecological restoration toward precise manipulation of microbial function. Proposed strategies include defined bacterial consortia, live biotherapeutic products, engineered microorganisms designed to produce beneficial metabolites, and bacteriophage-based interventions intended to suppress pathobionts without the collateral disruption caused by broad-spectrum antibiotics. Approaches of this type are particularly relevant in leukemia, where microbiome-associated effects involve epithelial barrier integrity, inflammatory regulation, and metabolic signaling, beyond taxonomic shifts alone (Nowicka et al., 2025; Cheng et al., 2025). These strategies align with broader precision-microbiome frameworks that integrate diet-based modulation, probiotics, prebiotics, postbiotics, FMT, engineered strains, phage-based approaches, and multi-omics-guided personalization, although their application in leukemia requires stricter safety validation because of neutropenia, mucosal injury, and immunosuppression (Bautista and López-Cortés, 2026a).

Pharmacomicrobiomic models add another dimension by suggesting that microbial composition and metabolic activity may influence drug metabolism, toxicity, therapeutic variability, and supportive care needs during leukemia treatment, although prospective validation in leukemia-specific cohorts remains limited. Microbiome-targeted interventions in this context extend beyond infection prevention or gastrointestinal recovery to include identification of patients at increased risk of adverse responses and refinement of supportive care strategies. Clinical implementation remains constrained by limited standardization, heterogeneous endpoints, and lack of leukemia-specific validation across treatment settings. Available data support continued development of function-oriented microbial therapeutics in hematologic malignancies (Le Ngoc et al., 2024; Hsu et al., 2026; Bautista et al., 2025c) (**Figure 1**).

## Limitations

Interpretation of microbiome data in leukemia is constrained by marked heterogeneity in cohort composition, disease subtype, and clinical exposure. Many available studies group biologically distinct leukemia entities or combine diagnostic and treatment phases, limiting the ability to define subtype-specific microbial patterns. This limitation is particularly relevant because AML, ALL, CLL, and CML differ in cellular origin, immune context, treatment intensity, age distribution, and duration of disease evolution. Consequently, findings derived from one subtype should not be generalized to leukemia as a whole without careful attention to clinical and methodological context. Most studies rely on small, single-center populations with substantial variation in age, treatment intensity, antibiotic exposure, nutritional support, hospitalization, and mucosal injury. Microbial alterations observed under these conditions cannot be attributed to leukemia alone, since multiple concurrent factors are known to reshape intestinal ecology (Gallant et al., 2025; Bautista et al., 2025a). In pediatric acute leukemia, interpretation is further complicated by the overlap between chemotherapy-induced perturbation, age-dependent microbiome maturation, repeated antimicrobial exposure, and prolonged supportive care (Rajagopala et al., 2020).

Sampling strategies introduce additional variability. Stool specimens are collected at different clinical stages, including diagnosis, induction therapy, post-antibiotic exposure, neutropenia, fever onset, and remission, each reflecting a distinct biological context. Persistent dysbiosis has been documented in children with ALL during chemotherapy and in adults with AML following intensive treatment; however, interpretation depends on sampling timing and concurrent exposures. Microbial configurations at AML diagnosis have been associated with baseline clinical features, although prior medication use, dietary variability, occult infection, and inflammatory stress remain difficult to control with precision (Jadhav et al., 2021; Rashidi et al., 2022).

Evidence remains predominantly associative. Reported associations between microbial disruption and clinical outcomes, including neutropenic fever, bloodstream infection, and mortality, do not resolve whether microbiota alterations participate in leukemogenesis or represent downstream consequences of disease and treatment (Rashidi et al., 2021). Leukemia modifies immune function, epithelial integrity, nutrient availability, and antimicrobial exposure, each capable of restructuring microbial communities. Dysbiosis may therefore represent a downstream effect, a modifier of disease trajectory, a marker of host vulnerability, or a clinical biomarker without necessarily acting as an initiating event (Peled et al., 2020; Goswami and Bose, 2024).

Functional interpretation remains limited by the scarcity of direct experimental validation. Associations between microbial composition, metabolite profiles, and host responses are increasingly reported, yet evidence demonstrating that specific taxa or microbial products directly alter leukemic biology remains insufficient. Altered microbiota-host metabolic interactions preceding neutropenic fever suggest biological relevance, although temporal proximity does not clarify whether microbial changes contribute to clinical deterioration or reflect pre-existing host vulnerability. Intestinal domination by selected taxa has been linked to subsequent bloodstream infection in transplant settings, but domination may result from antibiotic exposure, epithelial damage, and profound immunosuppression rather than mechanisms intrinsic to leukemogenesis. Strengthening causal inference will require longitudinal sampling frameworks, improved control of confounding variables, and experimental systems capable of testing direct effects on hematopoietic and leukemic phenotypes (Zhou et al., 2022; Habibi and Rashidi, 2023; Yang et al., 2024).

Reproducibility is affected by variability across cohorts, sequencing strategies, and analytical pipelines. Studies frequently combine pediatric and adult populations, distinct leukemia subtypes, and heterogeneous treatment stages, limiting generalizability of identified microbial patterns (Ece et al., 2026). Methodological differences further contribute to inconsistency. 16S rRNA sequencing provides limited taxonomic resolution and indirect functional inference, whereas shotgun metagenomics and integrative approaches offer broader characterization but are not uniformly applied. Variability in DNA extraction protocols, sequencing depth, contamination control, reference databases, and bioinformatic processing influences abundance estimates and downstream interpretation (Chakraborty et al., 2024).

Sample type introduces an additional limitation. Most data derive from stool, which incompletely represents mucosa-associated communities and does not capture microbial niches relevant in immunocompromised hosts. Differences in collection procedures, storage conditions, and processing intervals further affect comparability. Many studies are designed around endpoints such as infection, neutropenic fever, or treatment-related mortality rather than leukemogenesis itself (Liang et al., 2020; Jørgensen et al., 2025; Masetti et al., 2023). Associations identified in the context of treatment-related complications should therefore not be extrapolated to mechanisms of disease initiation or progression.

Integration of multi-omics data remains constrained by limited sample size, temporal misalignment, and analytical complexity. Although combining metagenomics, metabolomics, transcriptomics, proteomics, and clinical metadata has potential to improve biological resolution, available datasets in leukemia are often small and uneven across omics layers. Time-resolved integration is particularly challenging when molecular measurements are collected at non-overlapping time points, with substantial missing data and non-uniform preprocessing. In such settings, inferred interactions may lack stability, and temporal models may capture technical variation rather than biological relationships (Sherwani et al., 2025; Sibilio et al., 2025). Metabolite profiles in leukemia further reflect overlapping contributions from malignancy, chemotherapy, organ dysfunction, transfusion practices, nutritional support, and microbial activity, complicating attribution of specific effects (Liu et al., 2019).

Directionality remains unresolved even in integrative analyses. Correlations between taxa, metabolites, and clinical outcomes do not determine whether microbial alterations drive host metabolic changes, whether host physiology selects for specific microbial communities, or whether both processes are shaped by treatment and immune disruption. As a result, multi-omics studies in hematologic malignancies often generate descriptive interaction networks without establishing mechanistic frameworks with direct translational applicability (Zhang et al., 2023; Catalano et al., 2025).

Extensive clinical confounding, non-standardized sampling, limited causal resolution, and technical heterogeneity constrain interpretation. Although microbiome alterations are consistently observed in leukemia, their biological significance remains difficult to delineate due to overlapping effects of disease biology, treatment exposure, and host physiological instability (Theodosiou et al., 2026; Rajendran et al., 2024; Bautista et al., 2026c).

## Conclusions and future perspectives

Available evidence places the microbiome within the biological context in which leukemia develops, progresses, and is treated. Its contribution to disease initiation remains unresolved, but microbial alterations may be linked to leukemia biology, treatment tolerance, immune recovery, and therapy-related complications. Across independent cohorts, intestinal microbial alterations are consistently reported during leukemia progression, particularly in acute disease and under treatment exposure. Recurrent observations include reduced microbial diversity, depletion of SCFA-producing commensals, and expansion of opportunistic taxa such as *Enterococcus*. Despite these shared patterns, no microbial configuration reproducibly distinguishes leukemia across subtypes, age groups, or treatment contexts, indicating ecological imbalance without a disease-specific taxonomic signature (Ji et al., 2025; Zhou et al., 2022).

Associations linking microbiome variability with treatment-related outcomes are currently more consistent than evidence implicating microbial communities in leukemogenesis. In this setting, microbial features appear more closely related to treatment tolerance, infectious vulnerability, epithelial barrier injury, and host recovery than to upstream malignant transformation. Interpretation remains constrained by the predominance of observational human studies and by the unresolved directionality between dysbiosis, leukemia biology, and treatment-related disruption (Bautista et al., 2026b; Malard et al., 2025).

Experimental data provide mechanistic support for interactions between microbial ecosystems and hematopoietic regulation. Epithelial barrier dysfunction, microbial translocation, inflammatory signaling, immune remodeling, and altered metabolite availability converge as pathways through which microbial disruption can influence hematopoietic homeostasis. Evidence indicating that microbiota-derived metabolites promote ageing-associated clonal hematopoiesis through ALPK1 signaling further supports a functional connection between microbial products and pre-leukemic states. Progression from clonal hematopoiesis to overt leukemia, however, requires additional genetic and microenvironmental events that remain insufficiently defined (Agarwal et al., 2025; Zheng et al., 2024).

Interpretation of microbiome data in leukemia remains affected by multiple sources of variability. Antibiotic exposure, chemotherapy, hospitalization, nutritional changes, transfusion support, mucosal injury, and disease severity act concurrently on microbial composition, limiting attribution of observed alterations to leukemia itself. Additional heterogeneity arises from differences in cohort composition, sampling timepoints, sequencing strategies, taxonomic resolution, and specimen type. Divergent microbial findings across studies are therefore likely influenced, at least in part, by study design and technical variation rather than representing purely biological differences (Rashidi and Weisdorf, 2020; Jiang et al., 2025).

Advancement in this field depends on study designs capable of resolving temporal relationships and functional relevance with greater precision. Longitudinal, multicenter cohorts initiated prior to treatment and maintained across therapeutic phases are required to distinguish disease-associated microbial features from treatment-induced perturbations and to resolve whether microbial alterations precede clinically relevant events or reflect therapy and physiological instability. Integration of shotgun metagenomics, metabolomics, and host immune profiling will further enable identification of microbial functions linked to hematopoietic regulation beyond taxonomic composition (Nam et al., 2023; Sirasani et al., 2025). Future studies may also benefit from evaluating host-microbiome relationships as multi-compartment and function-oriented systems, as proposed in other oncology settings, while clearly distinguishing cross-cancer conceptual support from direct leukemia evidence (Bautista et al., 2026e).

During leukemia treatment, microbiome alterations are most relevant to supportive care. Loss of microbial diversity, reduced metabolic capacity, and intestinal domination by opportunistic organisms are associated with increased risk of infectious complications and delayed recovery under intensive therapy. Microbiome-informed risk stratification may therefore contribute to identifying vulnerable patients during treatment. Whether targeted microbiome interventions can modify clinical outcomes in a reproducible manner remains unresolved and requires controlled interventional studies in immunocompromised populations (Tian et al., 2025; Zhao et al., 2023).
